# Effect of Escitalopram on Glycemic Control and C-Reactive Protein in Patients With Depression and Co Morbid Type 2 Diabetes Mellitus – a Study on Indian Population

**DOI:** 10.1192/bjo.2024.693

**Published:** 2024-08-01

**Authors:** Shubhankar Tiwary, Vishnuvardhan G., Richa Tripathi, Manojprithviraj M.

**Affiliations:** 1 All India Institute Of Medical Sciences, Gorakhpur, India; 2 Rajarajeswari Medical College and Hospital, Bengaluru, India

## Abstract

**Aims:**

There is a bidirectional link between Depression and type 2 Diabetes mellitus (T2DM). Treatment of depression with selective serotonin reuptake inhibitors (SSRIs) may improve glycaemic control and may be beneficial for patients with comorbid depression and diabetes mellitus. The aim of the present study was to assess the effect of escitalopram on C-reactive protein (CRP) and glycaemic control in patients with comorbid T2DM and depression.

**Methods:**

A prospective interventional follow up study was conducted in a tertiary health care institute in urban India. Adult males and females who were diagnosed with Type 2 DM, having depression as per ICD-10 and treatment naïve for both the disorders were included for the study. Participants with other psychiatric disorders, on thyroid medication or on any medication that can have effect on CRP levels, having history of any infection/allergic or inflammatory conditions were excluded from the study. Sociodemographic details were collected. The severity of depression was assessed using Hamilton Depression Rating Scale (HDRS) at baseline. Escitalopram was started and titrated upto required doses for each patient. Levels of fasting blood glucose, post prandial blood glucose, HbA1c (Glycated Hemoglobin) and CRP were also measured at baseline. At the end of 3 months, severity of depression scores and blood levels of above mentioned parameters were measured and compared with their baseline values.

**Results:**

A total of 125 patients (females-n = 70, males-n = 55) were included for the study. The mean age of the sample was 63.2 years (SD 10.6). Most of the participants were educated and employed. The mean HRDS score of the participants at baseline and at three months was 20.3 (SD 3.7) and 18.0 (SD 3.9) respectively. The mean HbA1C of the participants at baseline and at three months was 8.4 (SD 1.2) and 7.8 (SD 1.2) respectively. The mean CRP of the participants at baseline and at three months was 4.0 (SD 5.6) and 2.8 (SD 4.3) respectively. There was significant reduction in depressive symptoms (Z score= -6.894, P value <0.05), levels of HbA1C (Z score= -7.936, P value <0.05) and CRP levels (Z score= -6.158, P value <0.05) at follow up after treatment with escitalopram. No significant correlation was observed in these parameters across gender.

**Conclusion:**

Treatment with escitalopram reduces the severity of depression and the ongoing inflammatory process amongst these patients.

